# Preterm birth and subsequent timing of pubertal growth, menarche, and voice break

**DOI:** 10.1038/s41390-021-01690-5

**Published:** 2021-08-24

**Authors:** Julia Suikkanen, Markku Nurhonen, Tim J. Cole, Marika Paalanne, Hanna-Maria Matinolli, Marjaana Tikanmäki, Marja Vääräsmäki, Marjo-Riitta Järvelin, Petteri Hovi, Eero Kajantie

**Affiliations:** 1 Department of Public Health and Welfare, Finnish Institute for Health and Welfare, Helsinki and Oulu, Finland; 2 Children’s Hospital, Pediatric Research Center, University of Helsinki and Helsinki University Hospital, Helsinki, Finland; 3 UCL Great Ormond Street Institute of Child Health, London, UK; 4 PEDEGO Research Unit, MRC Oulu, Oulu University Hospital and University of Oulu, Oulu, Finland; 5 Department of Child Psychiatry, University of Turku, Turku, Finland; 6 INVEST Research Flagship, University of Turku, Turku, Finland; 7 Children, Adolescents and Families Unit, Department of Welfare, Finnish Institute for Health and Welfare, Oulu, Finland; 8 Department of Epidemiology and Biostatistics, MRC–PHE Center for Environment & Health, School of Public Health, Imperial College London, London, UK; 9 Center for Life Course Epidemiology, Faculty of Medicine, University of Oulu, Oulu, Finland; 10 Biocenter Oulu, Oulu, Finland; 11 Unit of Primary Care, Oulu University Hospital, Oulu, Finland; 12 Department of Clinical and Molecular Medicine, Norwegian University of Science and Technology, Trondheim, Norway

## Abstract

**Background:**

We evaluated pubertal growth and pubertal timing of participants born preterm compared to those born at term.

**Methods:**

In the ESTER Preterm Birth Study, we collected growth data and measured final height of men/women born very or moderately preterm (<34 gestational weeks, *n* = 52/55), late preterm (34–<37 weeks, 94/106), and term (≥37 weeks, 131/151), resulting in median 9 measurements at ≥6 years. Timing of menarche or voice break was self-reported. Peak height velocity (PHV, cm/year) and age at PHV (years) were compared with SuperImposition by Translation And Rotation (SITAR) model (sexes separately).

**Results:**

Age at PHV (years) and PHV (cm/year) were similar in all gestational age groups. Compared to term controls, insignificant differences in age at PHV were 0.1 (95% CI: −0.2 to 0.4) years/0.2 (−0.1 to 0.4) for very or moderately/late preterm born men and −0.0 (−0.3 to 0.3)/−0.0 (−0.3 to 0.2) for women, respectively. Being born small for gestational age was not associated with pubertal growth. Age at menarche or voice break was similar in all the gestational age groups.

**Conclusions:**

Timing of pubertal growth and age at menarche or voice break were similar in participants born preterm and at term.

**Impact:**

- Pubertal growth and pubertal timing were similar in preterm and term participants in a relatively large cohort with a wide range of gestational ages.
- Previous literature indicates that small for gestational age is a risk for early puberty in term born children. This was not shown in preterm children.
- While our study had limited power for children born very preterm, all children born preterm were not at increased risk for early puberty.

## Introduction

Preterm birth (<37 gestational weeks) is a challenge for optimizing neonatal growth and may result in poor growth also during childhood.^1–6^ Most preterm born children catch up in weight but they often remain shorter than their term born peers,^4,5,7–9^ these changes being especially present in children born preterm and with small for gestational age birth weight or at earliest gestational weeks.^5,9–12^ Further, in some cohorts that comprise vulnerable groups of infants born at <26 gestational weeks, very preterm (<32 gestational weeks), or at very low birth weight (VLBW, <1500 g), preterm birth has been associated with a shorter adulthood stature.^6,9,10,13^

Among those born at term, lower birth weight (i.e., poorer fetal growth) predicts earlier age at menarche and earlier age of peak height velocity (PHV),^14–17^ and early puberty is associated with shorter adulthood stature.^18^ In general, children who mature early have higher PHV (cm/year) than late maturers.^19^ Literature is conflicting whether preterm birth is associated with timing of puberty or changes in pubertal growth pattern. In our previous cohort, the Helsinki Study of Very Low Birth Weight Adults (HeSVA), children born preterm and at VLBW had earlier age of pubertal growth acceleration and PHV compared to term controls.^20^ This difference was not affected by possible intrauterine growth retardation as it was seen in both appropriate for gestational age (AGA) and small for gestational age (SGA) participants.^20^ Another study detected advanced bone age at 12 years of age in VLBW children.^21^ On the other hand, one study that compared 129 preterm born children (<37 weeks) and 688 controls found no difference in pubertal growth spurt.^22^ When looking at other measures of pubertal timing, most preterm studies have found no differences in age of menarche, the majority of these reports coming from cohorts with a study group of VLBW or extremely low birth weight (ELBW) participants.^10,20,22–24^ Tanner staging was similar in two studies comparing preterm and VLBW or ELBW participants to those born at term at 14 or 15 years,^23,25^ but in a third study onset of puberty (assessed with Tanner staging) was later for preterm (<37 gestational weeks) girls than term controls.^26^ Possible problems at growth in utero and infancy, and catch-up growth later on, early life stress during neonatal intensive care unit (NICU) treatment, and tendency for insulin resistance already in childhood could be some of the programming factors (developmental origins) for possible earlier puberty among children born preterm.^14,27,28^

Our aim was to study pubertal growth, focusing on PHV (cm/year) and age at PHV (years), and timing of menarche and voice break in children born preterm across the range of preterm birth. We also explored differences between preterm children born either SGA or AGA. We hypothesized that preterm born children have earlier pubertal timing than children born at term.

## Methods

### Study participants

We invited 1980 young adults from Northern Finland, identified through the Northern Finland Birth Cohort 1986 (NFBC; born in 1985–1986; 49.8% of those invited) or the Finnish Medical Birth Register (FMBR; born in 1987–1989; 50.2%) to participate in the ESTER Preterm Birth Study.^29^ We conducted clinical examinations in 2009–2011 for 753 young adults at mean age of 23.3 (standard deviation (SD), 1.3) years: 149 very or moderately preterm (<34 gestational weeks), which has been referred to as early preterm in most previous publications from the ESTER Study), 248 late preterm (34–<37 gestational weeks), and 356 at term. The study was approved by the Coordinating Ethics committee, Helsinki and Uusimaa Hospital District, and all participants gave written informed consent.

Perinatal and postnatal data were previously collected from patient records for NFBC,^30^ and we collected the corresponding data for participants invited via FMBR.^29^ Gestational age was calculated from the last menstrual period or measured with ultrasonography (performed at <20 gestational weeks for 63% and 53% of fetuses to be born as preterm infants and controls, respectively).^29^ The diagnosis of gestational diabetes, gestational hypertension, and preeclampsia were set according to criteria previously described.^31,32^ SGA was defined as birth weight <−2 SD scores (SDSs) below mean birth weight for gestational age according to Finnish infant growth standards published in 1989,^33^ and other participants were defined as AGA.

In Finland, children’s height and weight are usually measured at least yearly until the age of 16 years in child welfare clinics or school health care. We collected heights, weights, and measurement dates from personal health records manually and measured adulthood height and weight in clinical visits at >19.9 years. Adulthood height was measured three times and the mean of these measurements was calculated. At the clinical visit, participants filled in a questionnaire including questions about menarche, voice break, and a self-evaluation of whether their pubertal timing was earlier or later than among peers. We excluded 15 participants with severe disability (severe mental disability, cerebral palsy, or severe physical disability) because these conditions may affect growth. We also excluded participants who had <2 height measurements available at ≥6 years of age (*n* = 149). All individual growth curves were visually inspected (J.S.) for outliers and 3 childhood height measurements were removed as incorrect. In total, the analysis included 589 participants: 52/55 men/women born very or moderately preterm (22/20 of them born <32 gestational weeks), 94/106 born late preterm, and 131/151 born at term. The median number of height measurements was 9 (interquartile range (IQR) 3) of which 5 (IQR 2) were performed during average pubertal years 9 and 16. Analyses included 16 participants with only 2–4 height measurements available.

### Data analysis

To compare characteristics of the gestational age groups, we used Student’s *t* test and Pearson’s chi-squared test. Differences of pubertal growth between preterm groups and controls were compared with the SuperImposition by Translation and Rotation (SITAR) growth curve model. SITAR is a nonlinear mixed effects model that calculates a fitted mean spline curve for the study group and individual differences in curve shape are explained by three parameters that the model estimates for each study participant: size, timing, and intensity.^34^ Each is expressed relative to the overall mean. The mean size, timing, and intensity of very or moderately preterm, late preterm, and term groups were compared separately for the two sexes. Geometrically on the height curve (age on *x* axis, height on *y* axis), size means translation on the *y* axis and is equal to difference of mean height in adulthood, timing means translation on the *x* axis and is equal to difference in age at PHV, and intensity means stretching or compressing the *x* axis and is equal to proportional difference of PHV from mean PHV.^34^ We present the differences of PHV (intensity parameter) in cm/year instead of proportional differences to ease interpretation of the results. The primary endpoints of this study were age at PHV (years) and PHV (cm/year). We included all height measurements at ≥6 years of age and the measurement age for adult height was set to 20 years (measured at >19.9 years). In our cohort, with 5 degrees of freedom for the mean curve as fixed effects, SITAR was able to explain >97% of the variance of pubertal growth, similar to previous studies.^34,35^

We present the data using chronological age (calculated from birth) because corrected age (calculated from due date) is usually only used clinically in children born very or moderately preterm for the first 2 or 3 years of life (recommended by World Health Organization and present Finnish growth charts)^36,37^ and because the relative difference between corrected and chronological age decreases during childhood.^5,10,25^ Additionally, we performed the main analyses with corrected age. Based on theoretical grounds, we also conducted the analyses with adjustments for source cohort, perinatal and postnatal characteristics (birth weight SDS, gestational diabetes, gestational hypertension or preeclampsia, maternal smoking during pregnancy, maternal age, maternal body mass index (BMI) before pregnancy), socioeconomic position (highest parental education at the time of adulthood examination), maternal height, and paternal height (reported by study participant, available for 84%). In addition, we conducted the analyses in predefined subgroups of very or moderately preterm and SGA, very or moderately preterm and AGA, late preterm and SGA, and late preterm and AGA, compared to those born at term. We also compared to controls separately those with birth weight <1500 g. To study the effect of prematurity as a linear variable, we performed the main analysis with gestational age as a continuous variable. R version 3.6.0 with package “sitar” and IBM SPSS Statistics 25 were used to perform the analyses and *p* < 0.05 was defined as the level of statistical significance.

## Results

### Nonparticipants

An analysis of those not attending the clinical study has been presented previously.^29^ In a nonparticipant analysis of those young adults who attended the adulthood assessment but had no childhood growth data available (*n* = 149 of total 753) compared with those who were included in the growth analyses, there was more maternal smoking (23% versus 18%) and maternal age was 1.0 year (95% confidence interval (CI): −2.1 to −0.0) less in these nonparticipants. Nonparticipants were 0.3 years (95% CI: 0.1 to 0.6) older at adulthood assessment than participants. Other characteristics did not differ between the two groups.

### Characteristics

As expected, very or moderately and late preterm born men and women differed from term born controls in their perinatal and postnatal characteristics (Table 1). Very or moderately and late preterm participants were on average 0.5 years (chronological age) (95% CI: −0.7 to −0.3) younger than term controls at adulthood assessment.

**Table 1:** Characteristics of the study participants.

	Very or moderately preterm^a^		Late preterm^a^		Term^a^		Very or moderately preterm/late preterm/term, *n*
	*n* (%) or mean (SD)		*n* (%) or mean (SD)		*n* (%) or mean (SD)		
	Men	Women	Men	Women	Men	Women	Missing
	*n* = 52	*n* = 55	*n* = 94	*n* = 106	*n* = 131	*n* = 151	
Gestational age, weeks	31.9 (1.9)*	32.2 (1.6)*	35.9 (0.8)*	35.8 (0.8)*	40.2 (1.1)	39.9 (1.3)	
Birth weight, g	1880 (480)*	1720 (440)*	2720 (480)*	2630 (540)*	3660 (490)	3480 (460)	
Birth weight SD score	−0.5 (1.4)*	−1.1 (1.3)*	−0.6 (1.2)*	−0.6 (1.3)*	−0.0 (1.0)	−0.0 (1.0)	
Small for gestational age^b^	6 (12%)*	13 (24%)*	11 (12%)*	14 (13%)*	2 (2%)	4 (3%)	
Birth length, cm	42.5 (3.1)*	41.9 (2.6)*	47.1 (2.3)*	46.3 (2.5)*	50.8 (2.1)	49.9 (1.8)	19/9/0
Birth length SD score	−0.2 (1.6)	−0.6 (1.4)*	−0.4 (1.2)	−0.4 (1.4)	−0.1 (1.1)	−0.1 (0.9)	19/9/0
Head circumference at birth, cm	30.3 (2.5)*	29.7 (2.2)*	33.4 (1.5)*	33.2 (1.5)*	35.5 (1.4)	34.8 (1.3)	28/26/2
Head circumference SD score at birth	0.3 (1.4)	−0.4 (1.3)	−0.1 (1.0)	0.1 (1.0)	0.0 (0.9)	−0.1 (0.9)	28/26/2
Twins or triplets	13 (25%)*	13 (24%)*	15 (16%)*	15 (14%)*	0 (0%)	3 (2%)	
Maternal smoking during pregnancy	6 (12%)	6 (11%)	13 (14%)	20 (19%)	23 (18%)	23 (15%)	7/5/5
Maternal hypertension without proteinuria (gestational or chronic)	6 (12%)	5 (9%)	15 (16%)	11 (10%)	13 (10%)	18 (12%)	0/5/4
Maternal preeclampsia	9 (17%)*	21 (38%)*	13 (14%)*	11 (10%)	7 (5%)	7 (5%)	0/5/4
Gestational diabetes	2 (4%)	2 (4%)	5 (5%)	4 (4%)	2 (2%)	3 (2%)	16/20/8
Mother’s age, years	30.0 (5.5)*	28.9 (5.3)	29.0 (5.9)	28.5 (6.2)	28.0 (5.8)	28.8 (5.4)	
Parental education							1/5/3
Lower secondary	4 (8%)	4 (7%)	9 (10%)	7 (7%)	5 (4%)	12 (8%)	
Upper secondary	29 (56%)	36 (65%)	50 (53%)	61 (58%)	84 (64%)	85 (56%)	
Lower tertiary	6 (12%)	4 (7%)	11 (12%)	16 (15%)	16 (12%)	17 (11%)	
Upper tertiary	12 (23%)	11 (20%)	23 (24%)	18 (17%)	24 (18%)	36 (24%)	
Mother’s body mass index, kg/m^2^	22.6 (3.2)	22.7 (3.9)	22.7 (3.7)	22.7 (4.1)	22.2 (3.2)	22.2 (3.0)	8/7/11
Mother’s height, cm	162.9 (5.4)	163.2 (5.7)	163.6 (5.2)	163.1 (4.9)	162.9 (5.4)	162.4 (5.5)	1/1/4
Father’s height, cm	177.2 (6.1)	175.7 (6.3)	176.8 (6.2)	177.9 (6.7)	176.5 (6.6)	176.8 (7.3)	19/35/42
Adult height, cm	178.1 (7.0)	163.7 (5.6)	178.0 (6.8)	164.2 (5.7)	177.7 (7.2)	163.9 (6.0)	
Adult weight, kg	76.3 (14.3)	64.8 (17.6)	78.9 (15.6)	63.2 (10.6)	77.0 (12.1)	62.2 (12.1)	
Adult BMI, kg/m2	24.0 (3.9)	24.1 (5.7)	24.9 (4.8)	23.4 (3.7)	24.4 (3.3)	23.2 (4.3)	
Age at adult assessment, years	22.9 (1.4)*	22.9 (1.3)*	23.1 (1.4)*	23.0 (1.2)*	23.6 (1.2)	23.4 (1.1)	

*Significant difference (*p* < 0.05) compared to controls of same sex, with Student’s *t* test or Pearson’s chi-squared test.

^a^Very or moderately preterm born <34 weeks, late preterm 34–<37 weeks, and term ≥37 weeks.

^b^Small for gestational age means birth weight <−2 SD according to Finnish growth charts (published in 1989).^33^

### Pubertal growth

**Fig. 1:**
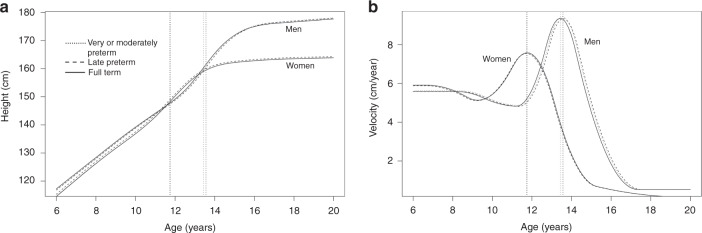
Summarized height and growth velocity curves. **a** SITAR growth curves for very and moderately preterm (<34 gestational weeks), late preterm (34–<37 gestational weeks) and term (≥37 gestational weeks) men and women. Vertical line demonstrates the age at peak height velocity. **b** Velocity of growth for very or moderately preterm, late preterm, and term men and women.

Age at PHV was 13.4 (SD 1.0) years (chronological age) in term born men and 11.8 (SD 0.9) years in term born women. The PHV (growth rate) (geometric mean and SD) was 9.4 (1.1) cm/year and 7.5 (1.1) cm/year in term born men and women, respectively. Preterm birth did not predict pubertal growth pattern: very or moderately and late preterm born men and women had similar adult height, age at PHV (years), and PHV (cm/year) (Table 2). Insignificant differences to term controls in age at PHV were 0.1 (95% CI: −0.2 to 0.4) years/0.2 (95% CI: −0.1 to 0.4) years for very or moderately/late preterm born men and −0.0 (95% CI: −0.3 to 0.3) years/−0.0 (95% CI: −0.3 to 0.2) years for women. The mean SITAR growth curves and velocity curves of all gestational age groups were closely similar, looking like a single curve for men and women as illustrated in Fig. 1. The differences remained statistically non-significant after repeating the analyses with corrected age (Supplemental Table S1 (online)). After adjusting the models for perinatal and postnatal factors and socioeconomic position, differences remained non-significant (Supplemental Table S2 (online)). Performing the analyses in subgroups according to birth weight (very or moderately preterm and SGA, very or moderately preterm and AGA, late preterm and SGA, late preterm and AGA, or VLBW, each compared with controls) showed no differences either; none of these groups differed from term controls at age at PHV (years) or PHV (cm/year) (Table 3). The small subgroup of VLBW men (*n* = 13) were 4.8 cm shorter in adult height than term born men, resulting also in a significantly different size parameter (−4.6 cm, 95% CI: -8.4 to −0.7), as expected (Table 3). In accordance with other analyses, gestational age as a continuous variable did not predict adult height, age at PHV (years), or PHV (cm/year) in men or women (Supplemental Table S3 (online)).

**Table 2:** Differences of adult height, age at peak height velocity (PHV), and PHV in very or moderately preterm and late preterm children compared to children born at term.

	Mean (SD) for term^a^ controls	Mean difference Very or moderately preterm^a^	Mean difference Late preterm^a^
		(95% CI)	(95% CI)
Adult height (cm)			
Men	177.7 (6.9)	0.5 (−1.7 to 2.7)	0.4 (−1.4 to 2.2)
Women	163.8 (5.8)	−0.1 (−1.8 to 1.7)	0.4 (−1.0 to 1.8)
Age at PHV (years)			
Men	13.4 (1.0)	0.1 (−0.2 to 0.4)	0.2 (−0.1 to 0.4)
Women	11.8 (0.9)	−0.0 (−0.3 to 0.3)	−0.0 (−0.3 to 0.2)
PHV^b^ (cm/year)			
Men	9.4 (1.1)	0.1 (−0.2 to 0.4)	0.0 (−0.3 to 0.3)
Women	7.6 (1.1)	−0.0 (−0.3 to 0.2)	−0.1 (−0.2 to 0.2)

^a^Very or moderately preterm born <34 weeks, late preterm 34–<37 weeks, and term ≥37 weeks.

^b^PHV was transformed to logarithms to attain normality and after analysis back-transformed to percentages and further to cm/year. The mean and SD for PHV are geometric mean and SD.

**Table 3:** Differences of adult height, age at PHV, and PHV in subgroups according to gestational age and birth weight compared to children born at term.

	Mean difference	Mean difference	Mean difference	Mean difference	Mean difference
	VLBW^a^	Very or moderately preterm^b^ and SGA^c^	Very or moderately preterm^b^ and AGA^d^	Late preterm^b^ and SGA^c^	Late preterm^b^ and AGA^d^
	(95% CI)	(95% CI)	(95% CI)	(95% CI)	(95% CI)
	13 men, 20 women	6 men, 13 women	46 men, 42 women	11 men, 14 women	83 men, 92 women
Adult height (cm)					
Men	−4.6 (−8.4 to −0.7)*	−4.0 (−9.5 to 1.5)	1.1 (−1.2 to 3.4)	−3.6 (−7.8 to 0.6)	1.0 (−0.9 to 2.8)
Women	−2.0 (−4.6 to 0.6)	−3.0 (−6.1 to 0.2)	0.6 (−1.3 to 2.5)	−3.2 (−6.2 to 0.1)	0.7 (−0.7 to 2.2)
Age at PHV (years)					
Men	0.1 (−0.5 to 0.6)	0.5 (−0.2 to 1.3)	0.1 (−0.3 to 0.4)	−0.0 (−0.6 to 0.5)	0.2 (−0.1 to 0.5)
Women	−0.1 (−0.5 to 0.4)	0.0 (−0.5 to 0.5)	−0.0 (−0.3 to 0.9)	−0.2 (−0.7 to 0.3)	−0.0 (−0.3 to 0.2)
PHV^e^ (cm/year)					
Men	0.0 (−0.5 to 0.6)	−0.6 (−1.3 to 0.2)	0.2 (−0.1 to 0.5)	−0.0 (−0.6 to 0.6)	−0.0 (−0.3 to 0.3)
Women	0.1 (−0.3 to 0.5)	−0.0 (−0.5 to 0.5)	−0.1 (−0.3 to 0.2)	−0.2 (−0.6 to 0.2)	−0.0 (−0.2 to 0.2)

*Significant difference (*p* < 0.05) compared to controls.

^a^Very low birth weight: preterm and <1500 g birth weight.

^b^Very or moderately preterm born <34 weeks, late preterm 34–<37 weeks, and term ≥37 weeks.

^c^Small for gestational age means birth weight <−2 SD according to Finnish growth charts (published in 1989).^33^

^d^Appropriate for gestational age means birth weight ≥−2 SD according to Finnish growth charts.^33^

^e^PHV was transformed to logarithms to attain normality and after analysis back-transformed to percentages and further to cm/year.

### Age of menarche and voice break and self-evaluation of pubertal timing

Very, moderately, and late preterm born women had similar mean age at menarche as controls (mean 12.7 years, SD 1.3), and very or moderately preterm men had similar age at voice break as term born men (mean 13.8 years, SD 1.2) (Table 4). Late preterm born men had their voice break 0.6 years (95% CI: 0.2 to 0.9) later than term born men, but when re-analyzed with the whole ESTER cohort (including 54 men born late preterm or term without growth data who had answered the questions about voice break), the difference was non-significant (*p* = 0.09). Self-assessment of pubertal timing was similar in all gestational age groups of men and women.

**Table 4:** Timing of menarche, voice break, and timing of puberty (self-evaluation).

	Very or moderately preterm	Late preterm	Term	n	Missing
	Mean (SD) or *n* (%)	Mean (SD) or *n* (%)	Mean (SD) or n (%)	Very or moderately preterm/late preterm/term	Very or moderately preterm/late preterm/term
*Age at menarche, years*	12.8 (1.3)	12.8 (1.2)	12.7 (1.4)	54/99/145	1/7/6
*Age at voice break, years*	13.6 (1.2)	14.4 (1.2)*	13.8 (1.2)	43/79/112	9/15/19
*Pubertal timing*				103/184/264	4/16/18
Earlier than average					
Women	10 (18.5)	18 (18.2)	31 (21.4)		
Men	8 (16.3)	11 (12.9)	18 (15.1)		
Average					
Women	34 (63.0)	69 (69.7)	91 (62.8)		
Men	34 (69.4)	65 (76.5)	82 (68.9)		
Later than average					
Women	10 (18.5)	12 (12.1)	23 (15.9)		
Men	7 (14.3)	9 (10.6)	19 (16.0)		

*Significant difference (*p* < 0.05) compared to controls of same sex, with Student’s *t* test or Pearson’s chi-squared test.

## Discussion

Very or moderately and late preterm born men and women had similar adult size, age at PHV (years), and PHV (cm/year) as term born controls. Accordingly self-reported age at menarche or voice break and self-evaluation of pubertal timing were similar in all gestational age groups. Confidence intervals were relatively narrow, meaning that we are able to exclude anything but small differences between groups. Several studies have shown that very preterm and VLBW children are smaller in childhood (both in height and weight) than their peers born at term, and they partly catch up during growth^5,8,10,25^ but in general remain shorter at final height.^6,7,10,13,38^ However, not many studies have reported childhood or pubertal growth or final height of children born late preterm. One study showed that moderately and late preterm children are lighter and shorter at 4 years of age than those born at term.^4^ In a large Swedish registry-based conscript study, those born moderately or late preterm (*n* = 9900) had the same BMI and 1 cm less adult height than term born men (analyses not adjusted for parental height),^39^ and another Swedish registry-based study showed that moderately preterm born women were 0.5 cm shorter than women born at term.^7^

Few studies have assessed timing of pubertal growth among children born preterm at a wide range of gestational ages. In a study that analyzed several perinatal factors, no differences emerged in timing of onset of pubertal growth in boys or girls born preterm (<37 gestational weeks, *n* = 129) compared to term controls (*n* = 688).^22^ By contrast, the HeSVA cohort showed earlier pubertal growth among children born preterm at VLBW: compared to term controls, VLBW SGA children (mean gestational age 31.7 weeks) reached PHV 5 months earlier (corrected age) and pubertal growth acceleration 11 months earlier (corrected age), and VLBW AGA children (mean gestational age 28.3 weeks) reached PHV 6 months earlier (corrected age) and acceleration 10 months earlier (corrected age).^20^ To replicate this analysis, we also compared preterm VLBW participants with controls but found no difference in timing of pubertal growth. However, this should be interpreted with caution as our study included only 33 VLBW participants.

A study with 2700 term born children showed a correlation with birth size (length and BMI) and timing of PHV in boys and girls, for example, in boys a 1 unit greater birth length SDS was associated with 0.11 years (standard error 0.03) later timing of PHV.^17^ In addition, 1 kg/m^2^ higher BMI at birth was associated with 0.05 years (standard error 0.023) later timing of PHV.^17^ These results indicate that smaller birth size predicts earlier timing of puberty.^17^ In our study, those born SGA had similar growth as controls, but, again, only 19 very or moderately preterm and 25 late preterm participants were born SGA.

Being a geographically based cohort, our study included also healthy infants born late preterm who did not need treatment in a NICU and thus were likely to be discharged from hospital early. Many infants of the cohort being healthy after birth may have protected them from the unfavorable early programming events (developmental origins of health and disease, Barker hypothesis) of prematurity^40^ and so not affected their pubertal timing. Also participants in the very or moderately preterm group were quite mature, with mean gestational age 32.0 weeks (SD 1.9), which may explain why our results differ from those of the HeSVA.^13,20^ Participants of the ESTER Study were born between 1986 and 1989, whereas the earlier HeSVA cohort recruited participants between 1978 and 1985, and this may have affected outcomes due to progress in neonatal medicine. The very or moderately preterm group in our study was also quite small, particularly those very preterm (22 men, 20 women). It is possible that the reasons why young adults chose to participate were different in the preterm group and the control group. For those born preterm, the reasons may have included gratitude to the health care system. If the control group were more interested in their physical capabilities, this may have attracted relatively more of them with a history of advanced puberty.

Some studies have measured timing of puberty using other indicators. In our current study, women born very or moderately preterm, late preterm, and at term reached menarche at a similar age (mean 12.7 years, SD 1.3). Several previous studies have also reported that VLBW girls reach menarche at the same age as those born at term.^10,22–25,41^ Likewise, men born very, moderately, or late preterm had similar age at voice break as term born men. Also in our previous HeSVA study, age at voice break was similar in men born at VLBW and at term.^20^ In both these studies, age at voice break was detected retrospectively. Few studies have presented Tanner staging (or measurement of testicular volume for boys) and no differences between VLBW or extremely low birth weight and term groups emerged at 14 or 15 years of age in two different studies.^23,25^ However, a study including some participants from the present study (the NFBC cohort), found very, moderately, and late preterm girls to be at an earlier pubertal stage (self-report at 16 years) than those born at term.^30^ A large study from Hong Kong showed preterm birth (including participants born <37 weeks) to be associated with a later onset of puberty in girls by Tanner staging.^26^ In the present study, the secondary outcome of pubertal timing, self-evaluation in young adulthood, did not differ between gestational age groups. However, self-report is far less accurate than repeated Tanner staging assessments before and during puberty.

Although the findings are conflicting, the majority of studies with different preterm groups have shown that preterm children go through developmental changes of puberty at the same pace as do children born at term. This is encouraging for the families of children born preterm. When looking at growth of preterm children in general, there can be big challenges for optimizing infant growth.^8,42^ In childhood, even moderately preterm children continue to be smaller (both in height and weight),^4^ but in puberty there seem not to be notable problems according to our findings. For teenagers themselves, developing normally and at the same time as others can have a large psychological lift. Earlier pubertal development has been associated with several harmful outcomes, including mental health problems, unhealthy relationships (adolescent dating abuse), and higher BMI in girls, and antisocial behavior, more sex partners, more drug use, and higher BMI in boys.^43–45^ Preterm birth alone creates a risk for internalizing mental health problems, metabolic abnormalities, and academic difficulties during childhood and adulthood, increasing the potential value of normal pubertal development for the health of people born preterm.^13,46–48^

The strengths of our study include a relatively large group of almost 600 participants (307 born preterm) with detailed growth data, median of 9 (IQR 3) height measurements at ≥6 years of age. Several studies have demonstrated great fit of the SITAR model during pubertal growth,^34,35,49^ which we used to investigate pubertal growth. Although reducing the characteristics of growth into three parameters, SITAR explained almost all individual differences of pubertal growth, >97% of the variance in this cohort. SITAR allows including participants with incomplete height data, which increases the number of participants in the analyses and increases the power of the study. The SITAR model is better than traditional linear growth curve models because of its nonlinear mean growth curve and its shifting age scale.^34^ Our cohort is geographically based with a wide variety in terms of condition of preterm infants after birth and, accordingly, a valuable setting for investigating epidemiological questions.

As to limitations, height and weight were measured as part of routine health check-ups in child welfare clinics and school health care, which did not include regular evaluation of pubertal development and Tanner staging. The growth data were collected retrospectively, so the practice for measuring height may have varied between centers, but all measurements were performed by trained nurses. The frequency of measurement was at least every other year for most participants, though some (*n* = 16, 3%) had only 2–4 measurements that may have affected the results. However, the SITAR model corrects for these uncertainties by weighting the more detailed growth curves. Age at menarche or voice break was asked retrospectively in young adulthood, which increases the risk of inaccuracy. Detailed nonparticipant analyses performed now and previously^29^ did not raise major concerns about participation bias, but it cannot be completely excluded.

To conclude, although preterm birth modifies growth in early childhood, children born across the range of prematurity presented similar pubertal growth (PHV and age at PHV) as their peers born at term. Preterm born children also attained menarche or voice break at the same mean age as term controls. These findings suggest that, at a population level, preterm birth is unlikely to have any meaningful association with pubertal timing or pubertal growth pattern.

## Supplementary information

Supplemental Table S1

Supplemental Table S2

Supplemental Table S3
