# Supplementary material for: Preterm birth and subsequent timing of pubertal growth, menarche, and voice break
Source: Pediatr Res. 2021 Aug 24;92(1):199–205. doi: 10.1038/s41390-021-01690-5 (PMC9411060; doi:10.1038/s41390-021-01690-5)
Supplement: Supplementary file 1 — Supplemental Table S1 [file 41390_2021_1690_MOESM1_ESM.docx]

Supplemental Table S1. Differences of adult height, age at peak height velocity (PHV), and PHV in very or moderately preterm and late preterm children compared to children born at term, with corrected ages^a^

|  | Mean difference Very or moderately preterm^b^  (95% CI) | Mean difference  Late preterm^b^  (95% CI) |
| --- | --- | --- |
| Adult height (cm),  Men | 0.5 (-1.7 to 2.7) | 0.4 (-1.4 to 2.2) |
| Women | -0.1 (-1.8 to 1.7) | 0.4 (-1.0 to 1.8) |
| Age at PHV (years), Men | -0.0 (-0.4 to 0.3) | 0.1 (-0.2 to 0.3) |
| Women | -0.2 (-0.4 to 0.1) | -0.1 (-0.3 to 0.1) |
| PHV^c^ (cm/year),  Men | 0.1 (-0.2 to 0.4) | 0.0 (-0.3 to 0.3) |
| Women | -0.0 (-0.3 to 0.2) | -0.1 (-0.2 to 0.2) |

^a^ Corrected age at term (40.0 gestational weeks) is 0.0 years

^b^ Very or moderately preterm born <34 weeks, late preterm 34 to <37 weeks and term ≥37 weeks

^c^ PHV was transformed to logarithms to attain normality and after analysis back-transformed to percentages and further to cm/year.
