# Supplementary material for: Preterm birth and subsequent timing of pubertal growth, menarche, and voice break
Source: Pediatr Res. 2021 Aug 24;92(1):199–205. doi: 10.1038/s41390-021-01690-5 (PMC9411060; doi:10.1038/s41390-021-01690-5)
Supplement: Supplementary file 2 — Supplemental Table S2 [file 41390_2021_1690_MOESM2_ESM.docx]

Supplemental Table S2. Differences of adult height, age at peak height velocity (PHV), and PHV in very or moderately preterm and late preterm children compared to children born at term in adjusted models

|  | Model^a^ | Mean difference Very or moderately preterm^b^  (95% CI) | Mean difference  Late preterm^b^  (95% CI) |
| --- | --- | --- | --- |
| Adult height (cm),  Men | 1 | 1.8 (-0.5 to 4.1) | 2.0 (0.2 to 3.9)* |
|  | 2 | 1.2 (-0.9 to 3.2) | 1.1 (-0.5 to 2.8) |
|  | 3 | 1.3 (-0.7 to 3.2) | 1.6 (-0.0 to 3.3) |
| Women | 1 | 1.2 (-0.7 to 3.1) | 1.5 (0.0 to 2.9)* |
|  | 2 | 0.3 (-1.5 to 2.1) | 0.9 (-0.4 to 2.2) |
|  | 3 | 0.7 (-0.2 to 2.6) | 0.9 (-0.5 to 2.2) |
| Age at PHV (years), Men | 1 | 0.2 (-0.2 to 0.5) | 0.3 (-0.0 to 0.5) |
|  | 2 | 0.1 (-0.2 to 0.5) | 0.2 (-0.1 to 0.5) |
|  | 3 | 0.1 (-0.3 to 0.4) | 0.3 (-0.1 to 0.6) |
| Women | 1 | 0.0 (-0.3 to 0.3) | 0.1 (-0.2 to 0.3) |
|  | 2 | -0.0 (-0.3 to 0.3) | 0.1 (-0.2 to 0.3) |
|  | 3 | -0.0 (-0.4 to 0.3) | 0.1 (-0.2 to 0.3) |
| PHV^c^ (cm/year),  Men | 1 | 0.1 (-0.2 to 0.5) | 0.0 (-0.2 to 0.3) |
|  | 2 | 0.1 (-0.3 to 0.4) | 0.0 (-0.3 to 0.3) |
|  | 3 | 0.1 (-0.3 to 0.5) | -0.1 (-0.4 to 0.3) |
| Women | 1 | -0.0 (-0.3 to 0.3) | -0.1 (-0.3 to 0.1) |
|  | 2 | -0.1 (-0.3 to 0.2) | -0.1 (-0.3 to 0.1) |
|  | 3 | -0.0 (-0.4 to 0.3) | -0.0 (-0.3 to 0.2) |

^*^ Significant difference (p < 0.05) compared to controls

^a^ Model 1: adjusted for cohort, birth weight SD-score, maternal smoking during pregnancy, maternal BMI, maternal age, gestational diabetes, maternal hypertension or preeclampsia, parental education.

Model 2: adjusted similar to Model 1 and also for maternal height

Model 3: adjusted similar to Model 2 and also for paternal height (missing information for 44 men and 42 women)

^b^ Very or moderately preterm born <34 weeks, late preterm 34 to <37 weeks and term ≥37 weeks

^c^ PHV was transformed to logarithms to attain normality and after analysis back-transformed to percentages and further to cm/year
