# Supplementary material for: Preterm birth and subsequent timing of pubertal growth, menarche, and voice break
Source: Pediatr Res. 2021 Aug 24;92(1):199–205. doi: 10.1038/s41390-021-01690-5 (PMC9411060; doi:10.1038/s41390-021-01690-5)
Supplement: Supplementary file 3 — Supplemental Table S3 [file 41390_2021_1690_MOESM3_ESM.docx]

Supplemental Table S3. Differences in adult height, age at peak height velocity (PHV), and PHV when gestational age increases one week

|  | Mean difference  (95% CI) |
| --- | --- |
| Adult height (cm),  Men | -0.03 (-0.26 to 0.21) |
| Women | 0.06 (-0.14 to 0.26) |
| Age at PHV (years), Men | -0.02 (-0.05 to 0.01) |
| Women | -0.00 (-0.03 to 0.03) |
| PHV^a^ (cm/year),  Men | -0.02 (-0.03 to 0.02) |
| Women | 0.02 (-0.02 to 0.04) |

^a^ PHV was transformed to logarithms to attain normality and after analysis back-transformed to percentages and further to cm/year
